# Leptin rs2167270 G > A (G19A) polymorphism may decrease the risk of cancer: A case‐control study and meta‐analysis involving 19 989 subjects

**DOI:** 10.1002/jcb.28378

**Published:** 2019-01-29

**Authors:** Jing Yang, Zhihui Zhong, Weifeng Tang, Jianping Chen

**Affiliations:** ^1^ Department of Gastroenterology The Third Affiliated Hospital of Soochow University Changzhou Jiangsu China; ^2^ Department of Orthopaedics Fuzhou Second Hospital Affiliated to Xiamen University Fuzhou Fujian China; ^3^ Department of Cardiothoracic Surgery Affiliated People’s Hospital of Jiangsu University Zhenjiang Jiangsu China

**Keywords:** leptin, meta‐analysis, polymorphism, risk

## Abstract

Accumulating evidence has suggested that leptin (LEP) is very important for the development of cancer. Recently, a number of case‐control studies about the relationship of the rs2167270 G > A (G19A) variants in the *LEP* gene with the risk of cancer have yielded inconsistent results. In this study, we have carried out a case‐control study [1063 esophagogastric junction adenocarcinoma (EGJA) cases and 1677 controls] in a Chinese population. Furthermore, we carried out a pooled‐analysis of 13 studies involving 8059 cancer patients and 11 930 controls to assess whether the *LEP* G19A locus was associated with overall cancer susceptibility. Odds ratios (ORs) with the corresponding 95% confidence intervals (CIs) were harnessed to evaluate the potential association. In our case‐control study, we found an association between the carriers of *LEP* 19A allele and EGJA risk. In addition, the results of meta‐analysis also suggested significant associations with cancer risk (A vs G: OR = 0.92, 95% CI = 0.88–0.97, *P* = 0.001; AA vs GG: OR = 0.83, 95% CI = 0.74–0.93, *P* = 0.001, GA/AA vs GG: OR = 0.93, 95% CI = 0.88–0.99, *P* = 0.023 and AA vs GG/GA: OR = 0.83, 95% CI = 0.74–0.92, *P* < 0.001). Upon conducting a stratified analysis, we found that *LEP* 19A allele might decrease the susceptibility of non‐Hodgkin lymphoma (NHL) and colorectal cancer (CRC). In a stratified‐by‐ethnicity analysis, significant associations were also found in Asians, Caucasians, and mixed populations. We can conclude that the *LEP* G19A polymorphism constitutes a decreased risk of cancer.

## 1 INTRODUCTION

Cancer is a major public health burden worldwide and has been the leading cause of death in China since 2010.^1^ Aging and unhealthy lifestyle (e.g. smoking, alcohol consumption, physical inactivity, and high fat, sugar and calorie diets) may contribute to the global burden of cancer.^2^, ^3^, ^4^ However, the carcinogenic effect is very complicated and remains unknown. Some studies reported that obesity, overweight, and type 2 diabetes may contribute to an individual’s cancer susceptibility.^5^, ^6^, ^7^

Leptin (LEP), a hormone of energy expenditure, may contribute to control energy expenditure and balance by suppressing hunger. LEP, a 16 kDa glycol‐protein, is predominantly made (>95%) by fat cells.^8^ LEP interacts with LEP‐receptor in the hypothalamus. A number of studies focused on the role of LEP in energy homeostasis and obesity. In addition, some investigations have demonstrated that LEP is associated with insulin signaling, inflammatory, and immune response.^9^, ^10^ Recently, several researchers reported that serum LEP levels might influence the development and progression of cancer.^11^, ^12^

It is found that the *LEP* rs2167270 G > A (G19A) locus is correlated with LEP levels and may also give a fascinating insight into the potential correlations with the development of cancer.^13^, ^14^ In a previous pooled study, it was found that individuals carrying a *LEP* 19A allele might have a lower tendency for cancer risk.^15^ However, most of the eligible studies focused on Caucasians. The potential relationship of this single‐nucleotide polymorphism (SNP) with cancer risk for Asians is unclear. Of late, several case‐control studies investigating the association between *LEP* G19A polymorphism and cancer risk have been performed in Asians with relatively large samples.^16^, ^17^ Thus, it may be meaningful to obtain data from additional studies to get a more meaningful assessment of genetic effects.

In this study, to acquire an understanding of the relationship between LEP polymorphism and risk of cancer, we first studied *LEP* G19A polymorphism with the susceptibility of developing esophagogastric junction adenocarcinoma (EGJA). And then, we performed a meta‐analysis to estimate the relationship of this polymorphism with overall cancer risk.

## 2 MATERIALS AND METHODS

### 2.1 Case‐control study

A total of 1063 unrelated EGJA cases were diagnosed and selected at Fujian Medical University Union Hospital, Fujian Medical University Cancer Hospital and Affiliated People’s Hospital of Jiangsu University. In addition, 1677 noncancer subjects were included in a control group. Both groups belonged to the Chinese Han populations from eastern China. The patients included 759 males and 304 females; the average age was 64.19 ± 8.63 years. Of them, 625 patients had lymph node metastases. There were 305 stage I/II and 758 stage III/IV EGJA patients included in the case group.^17^ The disease stage was assessed according to AJCC criteria (version 7.0). The control group was composed of 1194 males and 483 females with the mean age of 63.91 ± 10.22 years. Information regarding smoking and drinking has been described in our previous study.^17^, ^18^ Each participant signed a written informed consent. This study was approved by the review boards of the Jiangsu University as well as the Fujian Medical University. The genomic DNA was carefully extracted from peripheral venous blood of participants by using DNA Kit (Promega, Madison, Wisconsin). The *LEP* G19A polymorphism was detected by SNPscan genotyping assay (Genesky Biotechnologies Inc., Shanghai, China) according to conditions described by Chen et al.^17^

### 2.2 Meta‐analysis

We performed an extensive literature search in PubMed and EMBASE databases, covering all medical publications until 24 August 2018, with the following key words: LEP gene (e.g.: ‘LEP’ or ‘leptin’), cancer (e.g. ‘carcinoma,’ ‘cancer,’ ‘maligancy’ or ‘neoplasms’), and polymorphism (e.g.: ‘polymorphism,’ ‘SNP’ or ‘variation’). In addition, we also carried out a manual search of the listed references of the included publications and related reviews.

The criteria of literature selection were as follows: (a) investigation designed as a case–control study; (b) focusing on the association of *LEP* G19A polymorphism with risk of cancer; (c) genotypes data listing in the publications. The major exclusion criteria of studies were as follows: (a) reviews; (b) duplicated reports; (c) not case‐control study designs; (c) lack of data for genotype frequencies.

Two authors (J. Yang and Z. Zhong) extracted data from the included publications independently. The following information was collected: (a) first author; (b) publication year; (c) number of cases and controls; (d) country; (e) ethnicity; (f) source of controls; (g) cancer type; (h) genotyping method; and (I) genotype frequency. Ethnicities were defined as mixed, Asians, and Caucasians. For the source of controls, the publications were categorized as hospital‐based and population‐based studies.

In this study, we analyzed Hardy‐Weinberg equilibrium (HWE) using a goodness‐of‐fit test using an online software (https://ihg.gsf.de/cgi-bin/hw/hwa1.pl). The strength of the correlation between *LEP* G19A locus and cancer risk was determined by calculating crude odds ratios (ORs) with their 95% confidence intervals (95% CIs). The following four models were calculated: homozygote comparison (AA vs GG), dominant model (AA/GA vs GG), recessive model (AA vs GG/GA), and allele model (A vs G). If *I* ^*2*^ > 50% or *P* < 0.1, it suggested that there was significant heterogeneity. Considering the heterogeneity among the included studies, a different model was used to pool the data. When no significant heterogeneity was identified, the Mantel‐Haenszel method (fixed effects model) was used^19^; otherwise, the Der Simonian and Laird method (random model) was utilized.^20^, ^21^ Sensitivity analysis was also carried out, which deletes an individual investigation and, in turn, recalculates the remainders. The source of heterogeneity among variables (e.g. cancer type, ethnicity) was explored by subgroup analysis. Begg’s funnel plot and Egger’s regression method were harnessed to examine the publication bias among the included studies. And *P* < 0.1 was defined as representative of significant bias. The Newcastle‐Ottawa Quality Assessment Scale was used to assess the quality of the enrolled literatures. If scores ≥ 6 stars, the publication was considered as related high‐quality. In this study, all *P* values for statistics were calculated with two‐sided. STATA 12.0 software (Stata Corp, College Station, Texas) was used to analyze the data.

## 3 RESULTS

### 3.1 Case‐control study

A total of 2740 participants (involving 1063 EGJA patients and 1677 cancer‐free controls) were included in this case‐control study. Table 1 summarizes the primary information and our data for *LEP* G19A polymorphism.

**Table 1:** Primary information for *LEP* G19A polymorphism

Genotyped SNPs	MAF^a^ for Chinese in database	MAF in our controls (*N* = 1677)	*P* value for HWE^b^ test in our controls	Genotyping method	Genotyping value, %
*LEP* G19A	0.175	0.224	0.129	SNPscan	99.09

Abbreviation: SNP, single‐nucleotide polymorphism.

^a^ MAF: minor allele frequency.

^b^ HWE: Hardy‐Weinberg equilibrium.

Table 2 shows the genotype distributions of *LEP* G19A polymorphism. In the analysis of *LEP* G19A polymorphism, differences in the distribution of *LEP* G19A genotypes between EGJA patients and controls were found [GA vs GG: crude OR = 0.79, 95% CI = 0.67–0.93, *P* = 0.006; AA vs GG: crude OR = 0.57, 95% CI = 0.37–0.88, *P* = 0.012, GA/AA vs GG: crude OR = 0.79, 95% CI = 0.67–0.93, *P* = 0.004 and AA vs GG/GA: crude OR = 0.63, 95% CI = 0.41–0.97, *P* = 0.038]. The results of multivariate linear regression analysis also showed that *LEP* G19A polymorphism was correlated with a decreased risk of EGJA (GA vs GG: adjusted OR = 0.79, 95% CI = 0.67–0.93, *P* = 0.005; AA vs GG: adjusted OR = 0.58, 95% CI = 0.37–0.90, *P* = 0.015, GA/AA vs GG: adjusted OR = 0.79, 95% CI = 0.67–0.93, *P* = 0.004 and AA vs GG/GA: adjusted OR = 0.64, 95% CI = 0.41–0.99, *P* = 0.046).

**Table 2:** Logistic regression analyses of association between *LEP* G19A polymorphism and risk of EGJA

	Cases (*n* = 1063)		Controls (*n* = 1677)					
Genotypes	*n*	%	*n*	%	Crude OR (95% CI)	*P*	Adjusted OR^a^ (95%CI)	*P*
*LEP* rs2167270 G > A								
GG	678	65.13	998	59.62	1.00		1.00	
GA	334	32.08	603	36.02	**0.79(0.67‐0.93)**	**0.006**	**0.79(0.67‐0.93)**	**0.005**
AA	29	2.79	73	4.36	**0.57(0.37‐0.88)**	**0.012**	**0.58(0.37‐0.90)**	**0.015**
GA + AA	363	34.87	676	40.38	**0.79(0.67‐0.93)**	**0.004**	**0.79(0.67‐0.93)**	**0.004**
GG + GA	1012	97.21	1601	95.64	1.00		1.00	
AA	29	2.79	73	4.36	**0.63(0.41‐0.97)**	**0.038**	**0.64(0.41‐0.99)**	**0.046**
A allele	392	18.83	749	22.37				

Abbreviation: EGJA, esophagogastric junction adenocarcinoma.

Bold values are statistically significant (*P* < 0.05).

^a^ Adjusted for age, sex, smoking status, alcohol use and BMI status.

### 3.2 Meta‐analysis

**Figure 1:**
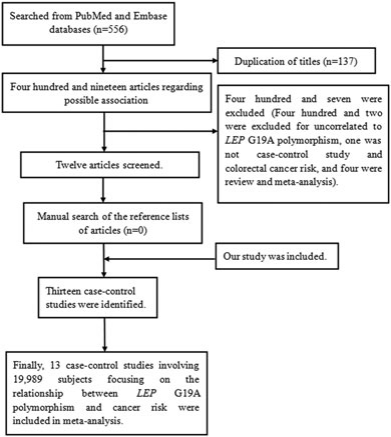
Flow diagram of the meta–analysis

We have summarized the meta‐analysis process in Figure 1. Finally, a total of 13 case‐control studies with 8059 cases and 11 930 controls were included in our analysis (Table 3). There were four case‐control studies and our investigation, conducted in Asian population,^16^, ^17^, ^22^, ^23^ six case‐control studies focused on Caucasian population,^24^, ^25^, ^26^, ^27^, ^28^, ^29^ and two case‐control studies performed in mixed population.^30^, ^31^ Tables 3 and 4 show the characteristics and genotyping data of the included studies, respectively. Table 5 demonstrate the process of quality assessment in this meta‐analysis.

**Table 3:** Characteristics of the studies in meta‐analysis

References	Publication year	Country	Ethnicity	Cancer type	Sample size (case/control)	Source of control	Genotype method
Skibola et al^24^	2004	USA	Caucasians	Non‐Hodgkin lymphoma	376/805	PB	TaqMan
Willett et al^25^	2005	UK	Caucasians	Non‐Hodgkin lymphoma	699/914	PB	TaqMan
Doecke et al^26^	2008	Australia	Caucasians	Esophageal cancer	774/1352	PB	Sequenom iPLEX
Slattery et al^30^	2008	USA	Mixed	Colorectal cancer	1565/1965	Mixed	TaqMan
Tsilidis et al^31^	2009	USA	Mixed	Colorectal cancer	208/381	PB	TaqMan
Wang et al^27^	2009	USA	Caucasians	Prostate cancer	258/258	PB	TaqMan
Moore et al^28^	2009	Finland	Caucasians	Prostate cancer	1053/1053	PB	TaqMan
Partida‐Perez et al^29^	2010	Mexico	Caucasians	Colorectal cancer	68/102	HB	PCR‐RFLP
Zhang et al^22^	2012	China	Asians	Non‐Hodgkin lymphoma	514/557	HB	TaqMan
Kim et al^23^	2012	Korea	Asians	Breast cancer	390/447	HB	MassARAY
Qiu et al^16^	2017	China	Asians	Esophageal cancer	507/1496	HB	SNPscan
Zhang et al^17^	2018	China	Asians	Hepatocellular carcinoma	584/923	HB	SNPscan
Our study	2018	China	Asians	Esophagogastric junction adenocarcinoma	1063/1677	HB	SNPscan

Abbreviations: HB, hospital‐based; PB, population‐based.

**Table 4:** Distribution of *LEP* G19A polymorphism genotype and allele

References	Publication year	Case AA	Case AG	Case GG	Control AA	Control AG	Control GG	Case A	Case G	Hardy‐Weinberg equilibrium
Skibola et al^24^	2004	36	169	168	119	335	351	241	505	No
Willett et al^25^	2005	79	276	235	122	357	275	434	746	Yes
Doecke et al^26^	2008	34	130	94	176	622	541	198	318	Yes
Slattery et al^30^	2008	190	766	611	304	867	794	1146	1988	No
Tsilidis et al^31^	2009	33	91	80	61	170	131	157	251	Yes
Wang et al^27^	2009	39	122	92	38	119	100	200	306	Yes
Moore et al^28^	2009	113	404	428	107	387	346	630	1260	Yes
Partida‐Perez et al^29^	2010	7	44	17	25	53	24	58	78	Yes
Zhang et al^22^	2012	26	166	322	29	190	338	218	810	Yes
Kim et al^23^	2012	12	110	269	18	147	284	134	648	Yes
Qiu et al^16^	2017	19	165	318	67	528	894	203	801	Yes
Zhang et al^17^	2018	34	198	343	36	321	564	266	884	Yes
Our study	2018	29	334	678	73	603	998	392	1690	Yes

**Table 5:** Quality assessment of the meta‐analysis

		Selection					Exposure			
References	Year	Adequate case definition	Representativeness of the cases	Selection of the controls	Definition of Controls	Comparability of the cases and controls	Ascertainment of exposure	Same ascertainment method for cases and controls	Nonresponse rate	Total Stars
Skibola et al^24^	2004	★	★	★	★	★★	★	…	…	7
Willett et al^25^	2005	★	★	★	★	★★	★	…	…	7
Doecke et al^26^	2008	★	★	★	★	…	…	…	…	4
Slattery et al^30^	2008	★	★	…	★	★★	★	…	…	6
Tsilidis et al^31^	2009	★	★	★	★	★★	★	…	…	7
Wang et al^27^	2009	★	★	★	★	★★	★	…	…	7
Moore et al^28^	2009	★	★	★	…	…	…	…	…	3
Partida‐Perez et al^29^	2010	★	★	…	★	…	★	…	…	4
Zhang et al^22^	2012	★	★	…	★	★★	★	…	…	6
Kim et al^23^	2012	★	★	…	★	★★	★	…	…	6
Qiu et al^16^	2017	★	★	…	★	★★	★	…	…	6
Zhang et al^17^	2018	★	★	…	★	★★	★	…	…	6
Our study	2018	★	★	…	★	★★	★	…	…	6

^★^means meet the standard

**Figure 2:**
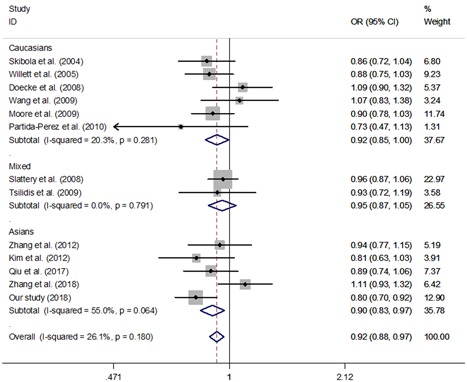
Meta‐analysis of the relationship between *LEP* G19A polymorphism and overall cancer risk (A vs G, fixed–effects model)

As demonstrated in Table 6, we identified a significant association of the G19A polymorphism in the *LEP* 5′‐UTR region with a decreased risk of overall cancer in four genetic models (A vs G: OR = 0.92, 95% CI = 0.88–0.97, *P* = 0.001; AA vs GG: OR = 0.83, 95% CI = 0.74–0.93, *P* = 0.001, GA/AA vs GG: OR = 0.93, 95% CI = 0.88–0.99, *P* = 0.023 and AA vs GG/GA: OR = 0.83, 95% CI = 0.74–0.92, *P* < 0.001, Figure 2). In this study, two studies were inconsistent with HWE.^24^, ^30^ When we excluded these studies, we also found that *LEP* G19A polymorphism decreased the risk of overall cancer (A vs G: OR = 0.92, 95% CI = 0.87–0.97, *P* = 0.002; AA vs GG: OR = 0.87, 95% CI = 0.75–0.99, *P* = 0.041 and GA/AA vs GG: OR = 0.90, 95% CI = 0.83–0.96, *P* = 0.003).

**Table 6:** Results of the meta‐analysis from different comparative genetic models

		A vs G				AA vs GG				AA + AG vs GG				AA vs AG + GG			
	No. of studies	OR (95% CI)	*P*	*I* ^*2*^	*P* (Q‐test)	OR (95% CI)	*P*	*I* ^*2*^	*P* (Q‐test)	OR (95% CI)	*P*	*I* ^*2*^	*P* (Q‐test)	OR (95% CI)	*P*	*I* ^*2*^	*P* (Q‐test)
Total	13	**0.92(0.88‐0.97)**	**0.001**	26.1%	0.180	**0.83(0.74‐0.93)**	**0.001**	25.9%	0.183	**0.93(0.88‐0.99)**	**0.023**	26.8%	0.174	**0.83(0.74‐0.92)**	**<0.001**	32.3%	0.124
HWE^a^	11	**0.92(0.87‐0.97)**	**0.002**	33.6	0.130	**0.87(0.75‐0.99)**	**0.041**	28.7	0.172	**0.90(0.83‐0.96)**	**0.003**	14.9%	0.302	0.89(0.78‐1.01)	0.080	24.7%	0.209
Ethnicity																	
Caucasians	6	**0.92(0.85‐1.00)**	**0.040**	20.3%	0.281	**0.82(0.70‐0.97)**	**0.022**	27.7%	0.227	0.94(0.84‐1.04)	0.237	4.6%	0.387	**0.83(0.71‐0.97)**	**0.017**	38.3%	0.151
Mixed	2	0.95(0.87‐1.05)	0.317	0.0%	0.791	**0.82(0.68‐1.00)**	**0.048**	0.0%	0.756	1.04(0.91‐1.18)	0.588	0.0%	0.330	**0.78(0.65‐0.93)**	**0.007**	0.0%	0.362
Asians	5	0.90(0.80‐1.02)	0.105	55.0%	0.064	0.87(0.61‐1.25)	0.452	55.8%	0.060	**0.87(0.79‐0.96)**	**0.005**	29.7%	0.223	0.91(0.65‐1.27)	0.571	48.8%	0.099
Cancer type																	
NHL	3	**0.89(0.80‐0.99)**	**0.025**	0.0%	0.819	**0.74(0.59‐0.94)**	**0.012**	0.0%	0.521	0.91(0.79‐1.04)	0.161	0.0%	0.875	**0.76(0.61‐0.94)**	**0.013**	0.0%	0.372
EC	2	0.98(0.80‐1.20)	0.834	57.7%	0.124	0.97(0.70‐1.35)	0.849	0.0%	0.335	1.00(0.74‐1.35)	0.999	67.1%	0.081	0.94(0.68‐1.28)	0.681	0.0%	0.581
CRC	3	0.94(0.86‐1.03)	0.205	0.0%	0.478	**0.80(0.66‐0.97)**	**0.023**	0.0%	0.381	1.03(0.91‐1.17)	0.620	0.0%	0.593	**0.75(0.63‐0.90)**	**0.002**	45.6%	0.159
PC	2	0.93(0.83‐1.06)	0.275	29.7%	0.233	0.91(0.70‐1.18)	0.485	0.0%	0.389	0.90(0.76‐1.06)	0.204	43.9%	0.182	0.96(0.75‐1.22)	0.740	0.0%	0.672
Others	3	0.90(0.72‐1.12)	0.341	76.9%	0.013	0.87(0.45‐1.68)	0.679	77.4%	0.012	0.87(0.71‐1.07)	0.194	63.5%	0.064	0.91(0.50‐1.67)	0.756	73.9%	0.022
Quality scores																	
≥6.0	10	**0.92(0.87‐0.97)**	**0.001**	25.8%	0.206	**0.82(0.72‐0.93)**	**0.001**	27.3%	0.192	**0.93(0.87‐0.99)**	**0.033**	28.0%	0.187	**0.81(0.72‐0.91)**	**<0.001**	28.8%	0.180
<6.0	3	0.94(0.84‐1.05)	0.280	49.2%	0.139	0.89(0.70‐1.13)	0.327	41.9%	0.179	0.94(0.81‐1.09)	0.429	26.8%	0.174	0.83(0.56‐1.23)	0.359	55.2%	0.107

Abbreviatons: CRC, colorectal cancer; EC, esophageal cancer; HWE, Hardy‐Weinberg equilibrium; NHL, non‐Hodgkin lymphoma; PC, prostate cancer.

Bold values are statistically significant (*P* < 0.05).

When an analysis stratified by cancer type was conducted, we found that individuals carrying *LEP* 19 A allele might have a lower susceptibility of NHL in three models (A vs G: OR = 0.89, 95% CI = 0.80–0.99, *P* = 0.025; AA vs GG: OR = 0.74, 95% CI = 0.59–0.94, *P* = 0.012 and AA vs GA/GG: OR = 0.76, 95% CI = 0.61–0.94, *P* = 0.013). In addition, we also found that the G19A polymorphism in *LEP* gene was correlated with a decreased susceptibility of CRC in homozygote comparison (OR = 0.80, 95% CI: 0.66‐0.97, *P* = 0.023) and recessive model (OR = 0.75, 95% CI: 0.63‐0.90, *P* = 0.002).

In an analysis stratified by ethnicities, significant associations were found also in Asians (GA/AA vs GG: OR = 0.87, 95% CI = 0.79–0.96, *P* = 0.005), Caucasians for three models (A vs G: OR = 0.92, 95% CI = 0.85–1.00, *P* = 0.040; AA vs GG: OR = 0.82, 95% CI = 0.70–0.97, *P* = 0.048 and AA vs GG/GA: OR = 0.83, 95% CI = 0.71–0.97, *P* = 0.017), and mixed population (AA vs GG: OR = 0.82, 95% CI = 0.68–1.00, *P* = 0.048 and AA vs GG/GA: OR = 0.78, 95% CI = 0.65–0.93, *P* = 0.007).

**Figure 3:**
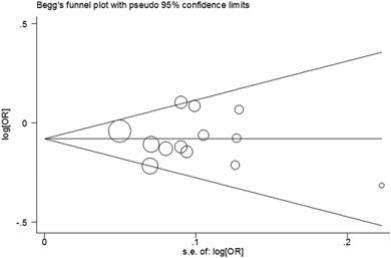
Begg’s funnel plot of meta–analysis (A vs G, fixed–effects model)

**Figure 4:**
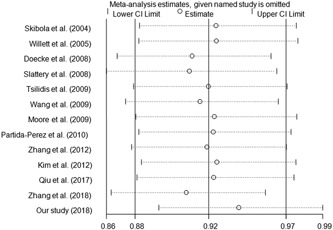
Sensitivity analysis of the influence of A vs G comparison (fixed–effects model)

We checked publication bias by using Begg’s funnel plot and Egger’s test. The statistical results showed that there was no significant bias in this meta‐analysis (A vs G: Begg’s test *P* = 1.00, Egger’s test *P* = 0.825; AA vs GG: Begg’s test *P* = 0.951, Egger’s test *P* = 0.975; GA/AA vs GG: Begg’s test *P* = 0.428, Egger’s test *P* = 0.981; AA vs GA/GG: Begg’s test *P* = 0.760, Egger’s test *P* = 0.847; Figure 3). One‐way sensitivity analysis was harnessed to confirm the stability of our findings. And we found that the corresponding results were not materially altered (Figure 4).

We assessed the quality score of the eligible studies by using the Newcastle‐Ottawa Quality Assessment Scale.^32^ The results are shown in Table 5. When the related low‐quality studies (<6.0) were excluded, the findings were not substantially changed (Table 6).

## 4 DISCUSSION

In this case‐control study, we found that *LEP* G19A polymorphism decreased the risk of EGJA. To the best of our knowledge, the first pooled‐analysis that carried out an extensive evaluation of the G19A polymorphism in the *LEP* 5′‐UTR region with the risk of overall cancer was conducted in 2014.^15^ In our meta‐analysis, 13 publications involving 8059 cases and 11 930 controls were included. Compared with the previous study, more new studies performed in Asian population were recruited.^16^, ^17^ Although some studies suggested that *LEP* G19A polymorphism could increase the risk of cancer,^17^ the pooled ORs of our study confirmed that G19A polymorphism in *the LEP* gene was correlated with a decreased risk of overall cancer. It is worth noting that this potential association was also observed in Caucasians, Asians, mixed populations, and NHL and CRC subgroups.

In the past few decades, some case‐control studies have been designed to explore the potential relationship between G19A polymorphism in the *LEP* gene and the risk of cancer.^16^, ^17^, ^22^, ^23^, ^24^, ^25^, ^26^, ^27^, ^28^, ^29^, ^30^, ^31^ Skibola et al^24^ found that *LEP* G19A polymorphism decreased the risk of NHL in Caucasians. Another study also identified similar findings regarding CRC in mixed populations.^30^ A previous meta‐analysis indicated that a tendency to decrease risk was noted between *LEP* G19A polymorphism and cancer.^15^ However, for Asian population, only two case‐control studies with small sample sizes were included in this pooled analysis.^22^, ^23^ The association of *LEP* G19A polymorphism with cancer risk in Asians was unclear. Recently, several studies investigated the relationship between *LEP* G19A polymorphism and cancer risk in Asians.^16^, ^17^ And they found no association between this SNP and cancer risk. Recently, Zhang et al^17^ reported that *LEP* G19A variants might increase the risk of HCC. The observed results were more controversial. In the current study, we conducted a case‐control study to identify the correlation between *LEP* G19A variants and the development of EGJA. We first found that *LEP* G19A polymorphism decreased the risk of EGJA in Asians. To estimate the relationship of *LEP* G19A polymorphism with cancer risk more extensively, we conducted an updated meta‐analysis. It was found that *LEP* G19A polymorphism may have a lower risk of overall cancer. *LEP* G19A polymorphism, a SNP in the 5′‐ untranslated region, could not be translated into amino acid peptides. However, this SNP may influence the RNA translation, stability, and transcription, and then alter the expression of LEP protein. A recent study indicated that *LEP* 19A allele is correlated with lower levels of LEP.^14^ A meta‐analysis found that the decreased serum LEP levels were a protective factor to breast cancer risk.^11^ It is conceivable that the reduced levels of serum LEP associated with *LEP* 19A allele may attenuate the risk of cancer. In this meta‐analysis, we confirmed this phenomenon. Furthermore, we identified a significant association in Caucasians and Asians for the first time.

The results of the heterogeneity analysis are shown in Table 6. We found that there was no evident heterogeneity across studies. Publication bias was evaluated by Begg’s funnel plots and Egger’s linear regression test. The results showed that no significant bias was observed. In this meta‐analysis, we assessed quality of the included studies. We found that the related low‐quality studies did not influence the findings of overall evaluation. These findings indicated that our conclusions were credible and stable.

Some limitations of the present pooled‐analysis should be acknowledged, even though it was powered by involving the latest literatures as well as our case‐control study. First, when the data were extracted and pooled, it was found that significantly heterogeneities existed among certain subgroups. Thus, these observed results should be explained with caution in these subgroups. Second, for the lack of critical data (eg such as age, sex, BMI, and environmental factors), gene‐environment interaction could not be carried out. Third, in this study, only *LEP* G19A polymorphism was studied; the interaction of gene‐gene was not evaluated. Fourth, in this study, a functional study focusing on the *LEP* G19A polymorphism was not conducted. Finally, because the eligible studies were limited, our analysis may be underpowered in some subgroups.

In conclusion, it is highlighted that the G19A polymorphism in the *LEP* 5′‐UTR region is associated with a decreased risk of EGJA. In addition, the subsequent meta‐analysis also indicates that this SNP decreases the risk of overall cancer. To confirm or refute our findings, large scale case‐control studies are needed.

## CONFLICTS OF INTEREST

The authors declare that they have no conflicts of interest.
